# Analysis of Best Practice Caregiving: A New Online Database of Evidence-Based Dementia Caregiving Programs

**DOI:** 10.1093/geroni/igab046.024

**Published:** 2021-12-17

**Authors:** Sara Powers, Sandy Markwood

## Abstract

Best Practice Caregiving (BPC) is a free online database providing comprehensive information on research and implementation characteristics for 44 evidence-based dementia caregiving programs. Programs eligible for BPC have research-tested positive outcomes for family/friend caregivers and demonstrated feasibility in community implementations. This symposium presents results from analyses of the BPC database that includes surveys of 44 program developers and 324 healthcare or community delivery-organizations, and content analysis of 231 published studies. Findings show the most common of 19 types of assistance provided by programs were: Supporting Caregiver/Individual-with-Dementia (IWD) Communication, Encouraging Positive Caregiver-IWD Activities, and Strengthening Coping (93.2%). Least common were: Getting a Dementia Diagnosis (29.5%) and Monitoring Service Benefits (20.5%). Methods of delivering the types of assistance were: information/referral (M=11.1), skills training (M=7.5), and direct provision of care (M=3.8). The most common types of organizations that delivered programs were healthcare organizations (23.8%) and Area Agencies on Aging (23.8%). The greatest delivery-challenges were program marketing (69.8%) and caregiver engagement (66.3%). Most organizations ‘strongly agreed’ that programs had positive impacts on caregivers (59.5%) but were less certain about IWD benefits (25.1% ‘strongly agreed’). Published research studies found the most improved caregiver outcomes were: 1) Strain and/or burden (84.1%), 2) Depressive symptomology (79.5%), and 3) Caregiving efficacy (63.6%). Least common improved outcomes were 1) Access to support information/Community service use (9.1%); 2) Unmet needs (6.8%); and 3) Respite/break from care (2.3%). Overall, results highlight strengths of evidence-based dementia caregiving programs, along with gaps and challenges to be addressed by existing and new developing programs.

